# Histone deacetylase inhibitors inhibit lung adenocarcinoma metastasis via HDAC2/YY1 mediated downregulation of Cdh1

**DOI:** 10.1038/s41598-023-38848-6

**Published:** 2023-07-26

**Authors:** Dongmei Wang, Yixiao Yang, Yuxiang Cao, Meiyao Meng, Xiaobo Wang, Zhengxun Zhang, Wei Fu, Shichao Duan, Liming Tang

**Affiliations:** 1 Department of Gastrointestinal Surgery, The Affiliated Changzhou, No. 2 People’s Hospital of Nanjing Medical University, Changzhou, 213004 Jiangsu China; 2 Changzhou Medical Center of Nanjing Medical University, Changzhou, 213004 Jiangsu China; 3 Institute of Burn Research, The First Affiliated Hospital, State Key Lab of Trauma, Burn and Combined Injury, Chongqing Key Laboratory for Disease Proteomics, Third Military Medical University (Army Medical University), Chongqing, 400038 China; 4 Shanghai Key Laboratory of Regulatory Biology, Institute of Biomedical Sciences and School of Life Sciences, East China Normal University, Shanghai, 200241 China; 5 Henan Provincial Chest Hospital, Zhengzhou, 450000 Henan China; 6 Henan Provincial People’s Hospital, Henan Eye Hospital, Henan Eye Institute, Zhengzhou University People’s Hospital, Henan University People’s Hospital, Zhengzhou, 450003 Henan China

**Keywords:** Lung cancer, Metastasis, Cancer, Cell biology

## Abstract

Metastasis is a leading cause of mortality in patients with lung adenocarcinoma. Histone deacetylases have emerged as promising targets for anti-tumor drugs, with histone deacetylase inhibitors (HDACi) being an active area of research. However, the precise mechanisms by which HDACi inhibits lung cancer metastasis remain incompletely understood. In this study, we employed a range of techniques, including qPCR, immunoblotting, co-immunoprecipitation, chromatin-immunoprecipitation, and cell migration assays, in conjunction with online database analysis, to investigate the role of HDACi and HDAC2/YY1 in the process of lung adenocarcinoma migration. The present study has demonstrated that both trichostatin A (TSA) and sodium butyrate (NaBu) significantly inhibit the invasion and migration of lung cancer cells via Histone deacetylase 2 (HDAC2). Overexpression of HDAC2 promotes lung cancer cell migration, whereas shHDAC2 effectively inhibits it. Further investigation revealed that HDAC2 interacts with YY1 and deacetylates Lysine 27 and Lysine9 of Histone 3, thereby inhibiting Cdh1 transcriptional activity and promoting cell migration. These findings have shed light on a novel functional mechanism of HDAC2/YY1 in lung adenocarcinoma cell migration.

## Introduction

Lung adenocarcinoma, the primary histological subtype of lung cancer, is responsible for the highest number of cancer-related fatalities globally^1^. The metastasis of lung adenocarcinoma is a perilous occurrence for individuals with cancer^2,3^. Epithelial-mesenchymal transition (EMT) contributes to tumor-invasive phenotypes and metastasis of lung adenocarcinoma^4,5^

Histone acetylation is a prevalent and significant epigenetic regulatory mechanism that is closely associated with tumorigenesis. It is primarily governed by histone acetyltransferases (HATs) and histone deacetylases (HDACs)^6^. The dynamic and reversible nature of this mechanism renders key enzymes as ideal targets for drug intervention^7,8^. HDAC inhibitors (HDACi) have been shown to elevate the acetylation level in the promoter region of specific genes, thereby augmenting the expression of related genes^9^. Several preclinical investigations have demonstrated the remarkable antitumor efficacy of HDAC inhibitors in lung cancer cell lines^10^. Nevertheless, the precise mechanisms underlying the inhibition of lung cancer metastasis by HDACi remain incompletely understood. Mammalian cells contain four classes of HDACs^11^, and it is imperative to identify the principal HDACs implicated in the progression of lung adenocarcinoma. This study discloses that HDAC2 serves as the pivotal deacetylase in TGF-β-induced EMT and cell migration.

Histone deacetylases (HDACs) have the ability to modify chromatin structure and DNA accessibility by altering the acetylation levels of histones^12^, this process can result in significant changes in fundamental mechanisms such as cell proliferation, cell-cycle progression, apoptosis, and epithelial-to-mesenchymal transition^13,14^. Specifically, HDAC2 serves as a crucial epigenetic regulator of gene expression in cancer^15^. Research has demonstrated that HDAC2 plays a pivotal role in regulating the cell cycle and apoptosis in both normal and tumor cells^16^. Furthermore, HDAC2 has been linked to the modulation of diverse signaling pathways, such as TGF-β and NF-κB, which are implicated in asthma and chronic obstructive pulmonary disease^17^. Furthermore, studies indicate that HDAC2 plays a role in the self-renewal^18^, differentiation, and motility^19^ of cancer stem cells. Collectively, these findings underscore the pivotal role of HDAC2 in cancer biology. As such, comprehending the precise mechanisms underlying the functions of Histone Deacetylase 2 (HDAC2) in cancer is a crucial stride towards devising innovative therapeutic approaches for cancer management.

Yin Yang 1 (YY-1) is a zinc finger protein that is classified as a GLI-Kruppel transcription factor^20,21^ and is expressed ubiquitously in mammalian cells. Its regulatory function encompasses both transcriptional activation and repression, which appears to be in a context-dependent manner^22^. Specifically, YY1 is capable of directing histone deacetylases and histone acetyltransferases to a promoter site, thereby activating or repressing the promoter. This suggests that histone modification plays a role in YY1's regulatory function.

The present study aimed to examine the involvement of HDACi in TGF-β-mediated cell migration and ascertain the significance of HDAC2 in lung adenocarcinoma cell migration by means of its interaction with YY1 and repression of EMT-related molecule transcriptional activity. The findings of this investigation unveil a novel role of HDAC2/YY1 in lung adenocarcinoma migration.

## Results

### HDAC2 is involved in HDAC inhibitors suppressed TGF-β-induced EMT of lung adenocarcinoma cells

**Figure 1:**
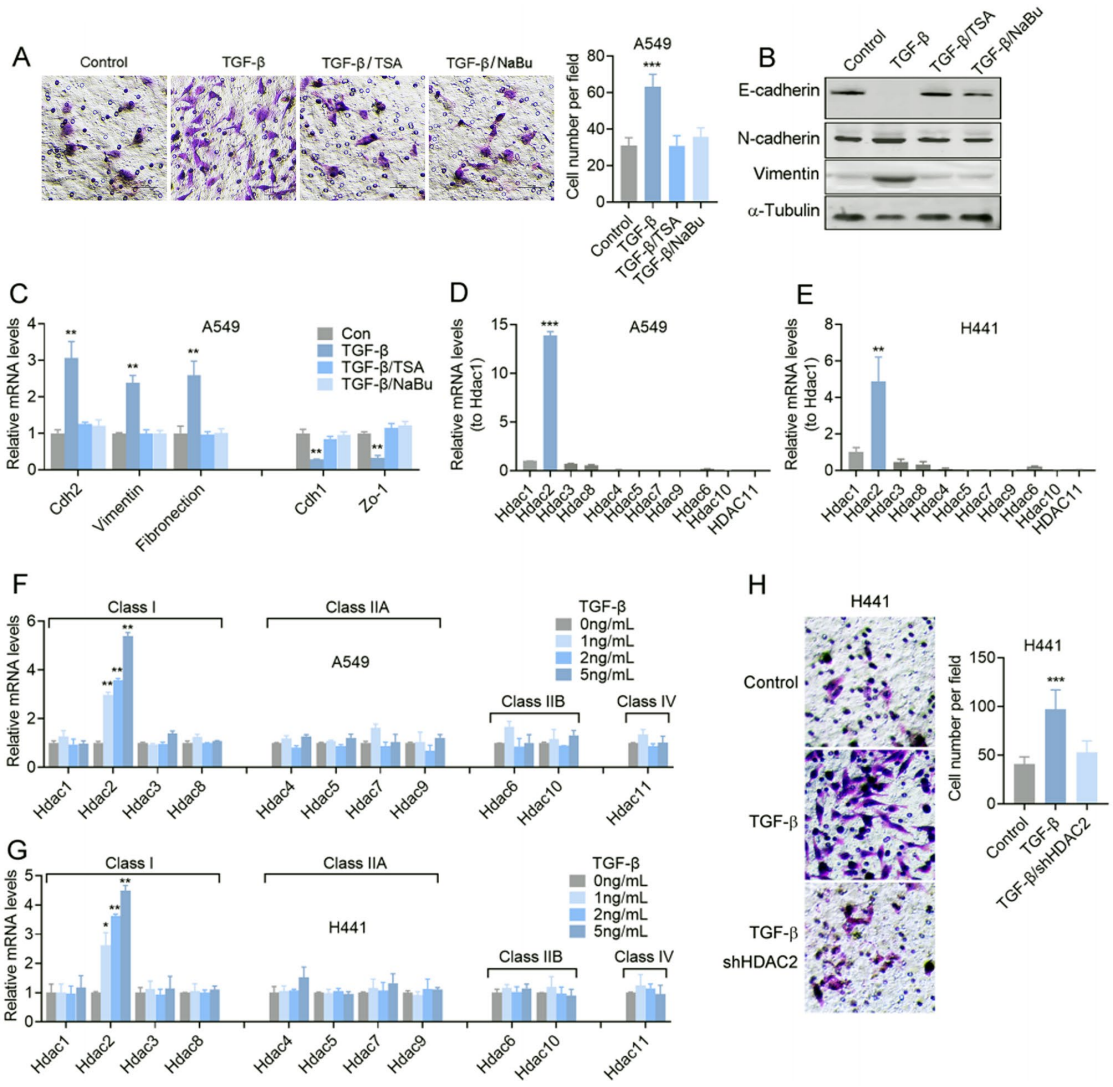
HDAC inhibitors suppress TGF-β-induced EMT of lung adenocarcinoma cells via HDAC2. (**A**) Transwell assay and (**B**) immunoblotting of A549 lung adenocarcinoma cells pretreated with 5 ng/mL TGF-β in the presence of 20 ng/mL TSA or 2 mM NaBu for 48 h. (**C**) Relative mRNA levels of EMT related molecules in A549 cells pretreated with 5 ng/mL TGF-β in the presence of 20 ng/mL TSA or 2 mM NaBu for 24 h. Relative mRNA levels of Hdacs in A549 (**D**) and H441 (**E**) lung adenocarcinoma cells. Relative mRNA levels of Hdacs in A549 (**F**) or H441 (**G**) cells pretreated with different doses of TGF-β for 24 h. (**H**) Transwell assay of shHDAC2 A549 cells pretreated with 5 ng/mL TGF-β. Magnification is 200-fold, and the scale bar is 50 μm. Data are presented as mean ± SEM, and **P < 0.01, ***P < 0.001 compared with the control group. All experiments were performed at least three times.

In order to investigate the potential involvement of HDACs in the migration of lung cancer cells, we conducted an examination of the effects of two HDAC inhibitors, trichostatin A (TSA) and sodium butyrate (NaBu), which are structurally unrelated. Specifically, we sought to determine the impact of these inhibitors on TGF-β-induced EMT in A549 and H441 lung adenocarcinoma cells. Our findings indicate that both HDAC inhibitors effectively prevented TGF-β-induced cell migration (Fig. 1A) and EMT (Fig. 1B,C and Fig. S1A). Given that TSA and NaBu are pan-HDAC inhibitors, we further sought to identify the most relevant HDACs involved in this process by examining the expression of different HDACs in lung adenocarcinoma cells. We found that HDAC2 exhibits the highest level of expression among all HDACs (Fig. 1D,E), and its expression is positively correlated with the dosage of TGF-β (Fig. 1F,G). We also found that TSA and NaBu have no obvious effects on the mRNA change of TGF-β induced the expression of HDACs (Fig. S1B). Furthermore, our findings indicate that HDAC2 knockdown effectively inhibits H441 cell migration with or without TGF-β treatment (Fig. 1H and Fig. S1C, D), implying that HDAC2 may be involved in the process of TGF-β-induced EMT. In conclusion, our study highlights the significant role of HDAC2 in TGF-β-induced EMT and suggests that HDAC2 could be a promising therapeutic target for the inhibition of lung cancer cell migration.

### HDAC2 is highly expressed in lung adenocarcinomas cells

**Figure 2:**
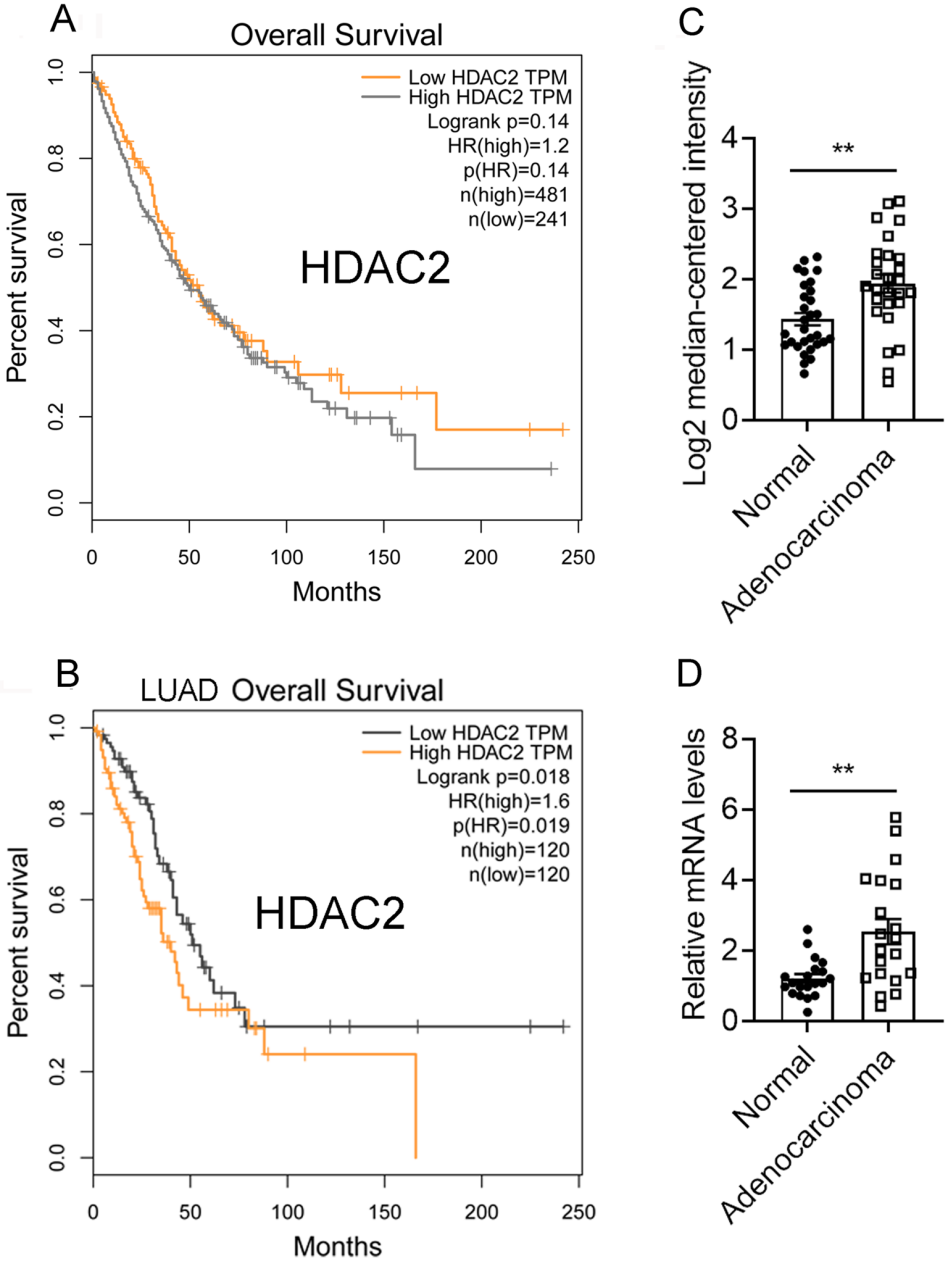
The correlation of HDACs expression with lung cancer patients’ survival. (**A**) GEPIA showing the correlation between HDAC2 expression levels and the overall survival of lung cancer patients from the TCGA database. (**B**) GEPIA showing the correlation between HDAC2 and the overall survival of lung adenocarcinoma patients from the TCGA database. (**C**, **D**) The expression of HDAC2 in lung adenocarcinoma and lung tissues from the *Su* lung database (**C**) and clinical lung adenocarcinoma and lung tissues (**D**). Data are presented as mean ± SEM, and **P < 0.01.

In order to examine the potential impact of HDAC2 on the survival of individuals with lung cancer, an analysis of the TCGA database was conducted utilizing GEPAI. The results indicated that the expression of HDAC2 was not significantly associated with the overall survival of lung cancer patients (Fig. 2A). Nevertheless, a negative correlation was observed between the expression of HDAC2 and the overall survival of lung adenocarcinoma patients (Fig. 2B), which suggests that HDAC2 may play a role in the advancement of lung adenocarcinoma.

Further analysis of the TCGA database demonstrated a marked elevation of HDAC2 expression in lung adenocarcinomas (Fig. 2C). Our additional scrutiny of HDAC2 expression in clinical lung adenocarcinomas corroborated this observation (Fig. 2D). These findings provide evidence that HDAC2 is significantly upregulated in lung adenocarcinoma cells and is closely associated with the overall survival of lung adenocarcinoma patients, suggesting that HDAC2 may exert a pivotal role in the progression of lung adenocarcinoma and could represent a promising therapeutic target.

### HDAC2 promotes lung adenocarcinomas migration

**Figure 3:**
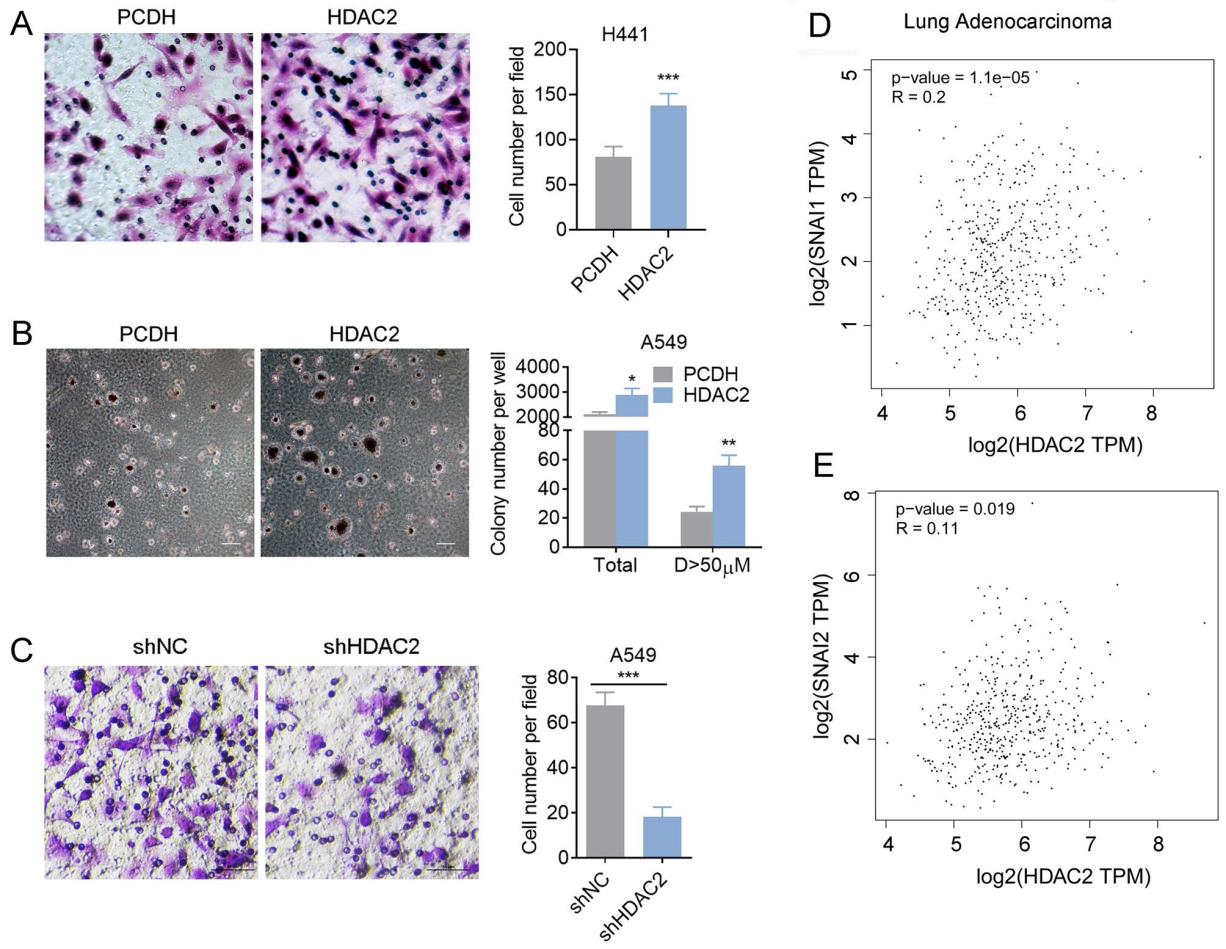
HDAC2 promotes lung adenocarcinoma migration. (**A**) Transwell assay of NC or HDAC2 overexpressed H441 cells. (**B**) Colony formation assay of NC or HDAC2 overexpressed A549 cells. (**C**) Transwell assay of NC or shHDAC2 A549 cells. (**D**, **E**) The correlation of HDAC2 with EMT-related transcription factors. Magnification is 200-fold, and scale bar is 50 μm. Data are presented as mean ± SEM, and *P < 0.05, **P < 0.01, ***P < 0.001 compared with the control group. All experiments were performed at least three times.

In order to examine the involvement of HDAC2 in the migration of lung adenocarcinoma cells, we conducted an overexpression of HDAC2 in A549 and H441 cells, both are well used KRAS-mutant lung adenocarcinoma. Our results indicate that HDAC2 facilitates cell migration (Fig. 3A,B and Fig. S2A) and metastasis (Fig. S2B). To further validate the role of HDAC2, we also assessed the impact of HDAC2 knockdown on cell migration (Fig. 3C and Fig. S2C). Additionally, we conducted a thorough analysis of the TCGA database, which revealed a positive correlation between HDAC2 and key EMT-related transcription factors, such as snail1 and snail2, but not other transcriptional factors, in lung cancer tissues (Fig. 3D,E and Fig. S2C). These findings suggest that HDAC2 plays a crucial role in promoting lung adenocarcinoma metastasis.

### HDAC2 interacts with YY1 in lung adenocarcinoma cells

**Figure 4:**
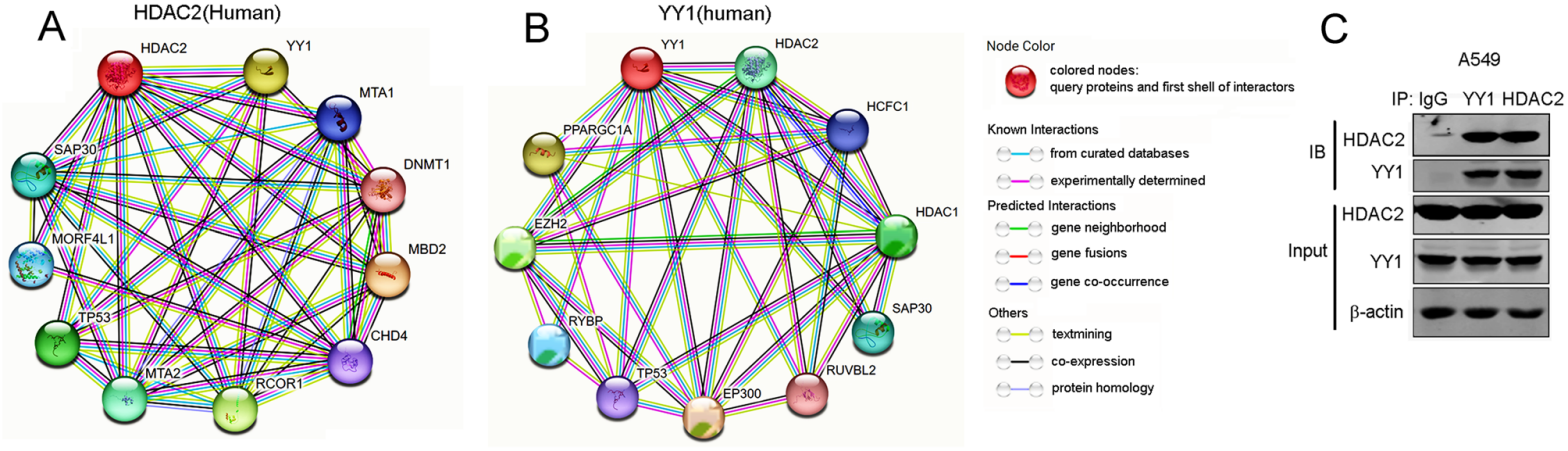
HDAC2 interacts with YY1. (**A**) The top ten proteins interacted with HDAC2 analyzed by STRING. (**B**) The top ten proteins interacted with YY1 analyzed by STRING. (**C**) Immunoblotting and co-immunoprecipitation of endogenous HDAC2 and YY1 in A549 cells. All experiments were performed at least three times.

In order to explicate the functional mechanism of HDAC2 in the cellular migration process, an analysis of its interaction proteins was conducted using STRING. This analysis revealed the presence of a well-known tumor inducer, Yin Yang 1 (YY-1) (Fig. 4A). Further analysis of YY-1’s interaction proteins indicated that HDAC2 was among its top 10 binding proteins (Fig. 4B). To delve deeper into this interaction in the context of lung adenocarcinoma, the interaction of endogenous HDAC2 and YY1 was examined in A549 cells, revealing that YY1 and HDAC2 can indeed interact with one another (Fig. 4C and Fig. S3). These findings suggest that HDAC2 has the potential to interact with YY1.

### YY1 functions as a collaborative partner with HDAC2 to facilitate cellular migration

**Figure 5:**
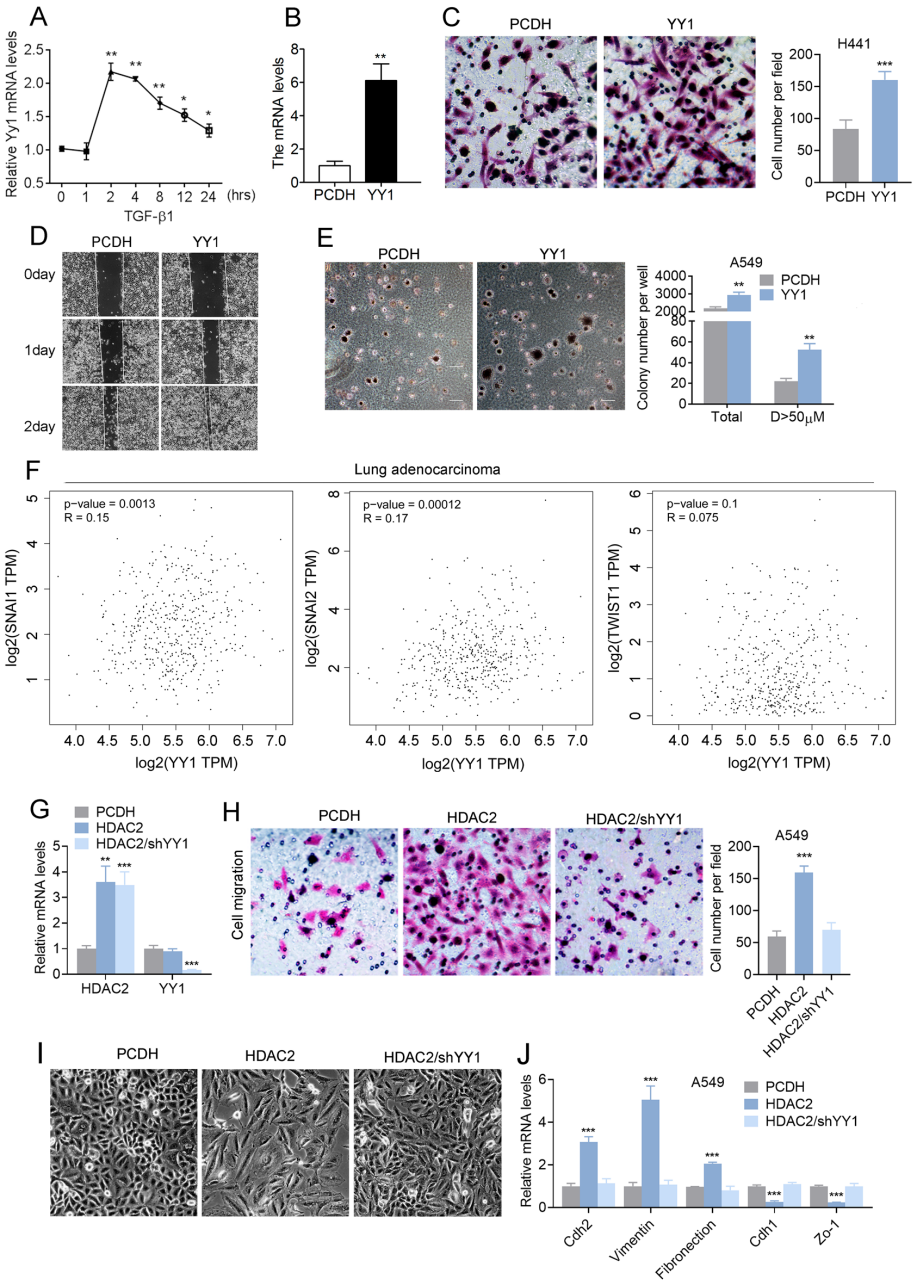
HDAC2 promotes cell migration dependent on YY1. (**A**) The mRNA level of YY1 in TGF-β induced EMT in A549 cells. (**B**) The mRNA level and (**C**) transwell assay of YY1-overexpressed H441 cells. (**D**) The wound healing and (**E**) colony formation assay of YY1-overexpressed A549 cells. (**F**) The correlation of HDAC2 with EMT-related transcription factors. (**G**) The mRNA level of HDAC2 and YY1, (**H**) transwell assay, (**I**) cell morphological change, and (**J**) the mRNA level of EMT markers of HDAC2-overexpressed A549 cells in the absence of YY1 (shYY1). Magnification is 200-fold, and scale bar is 50 μm. Data are presented as mean ± SEM, and *P < 0.05, **P < 0.01, ***P < 0.001 compared with the control group. All experiments were performed at least three times.

The identification of HDAC2's interaction with YY1 in lung adenocarcinoma cells prompted an investigation into the involvement of YY1 in lung adenocarcinoma migration. Our findings indicate that TGF-β induced YY1 expression within 2 h of treatment, followed by a gradual decrease until 24 h (Fig. 5A,B and Fig S4A, B). Moreover, the overexpression of YY1 significantly facilitated cell migration (Fig. 5C–E and Fig. S4C) and lung metastasis (Fig. S4D). Further analysis revealed a positive correlation between YY1 and key EMT-related transcription factors, such as snail1 and snail2, but not other transcriptional factors (Fig. 5F).

In order to validate the crucial involvement of YY1 in HDAC2-mediated cellular migration, we conducted a deletion of YY1 in HDAC2 overexpressed lung adenocarcinoma cells (Fig. 5G). Our findings demonstrate that the removal of YY1 resulted in the elimination of HDAC2-induced cellular migration (Fig. 5H–J). These outcomes provide evidence that YY1 serves as a functional collaborator with HDAC2 in the migration of lung adenocarcinoma cells, and emphasize the potential for targeting HDAC2-YY1 interactions as a means of developing innovative therapeutic approaches.

### YY1 and HDAC2 is highly expressed in lung adenocarcinoma tissues

**Figure 6:**
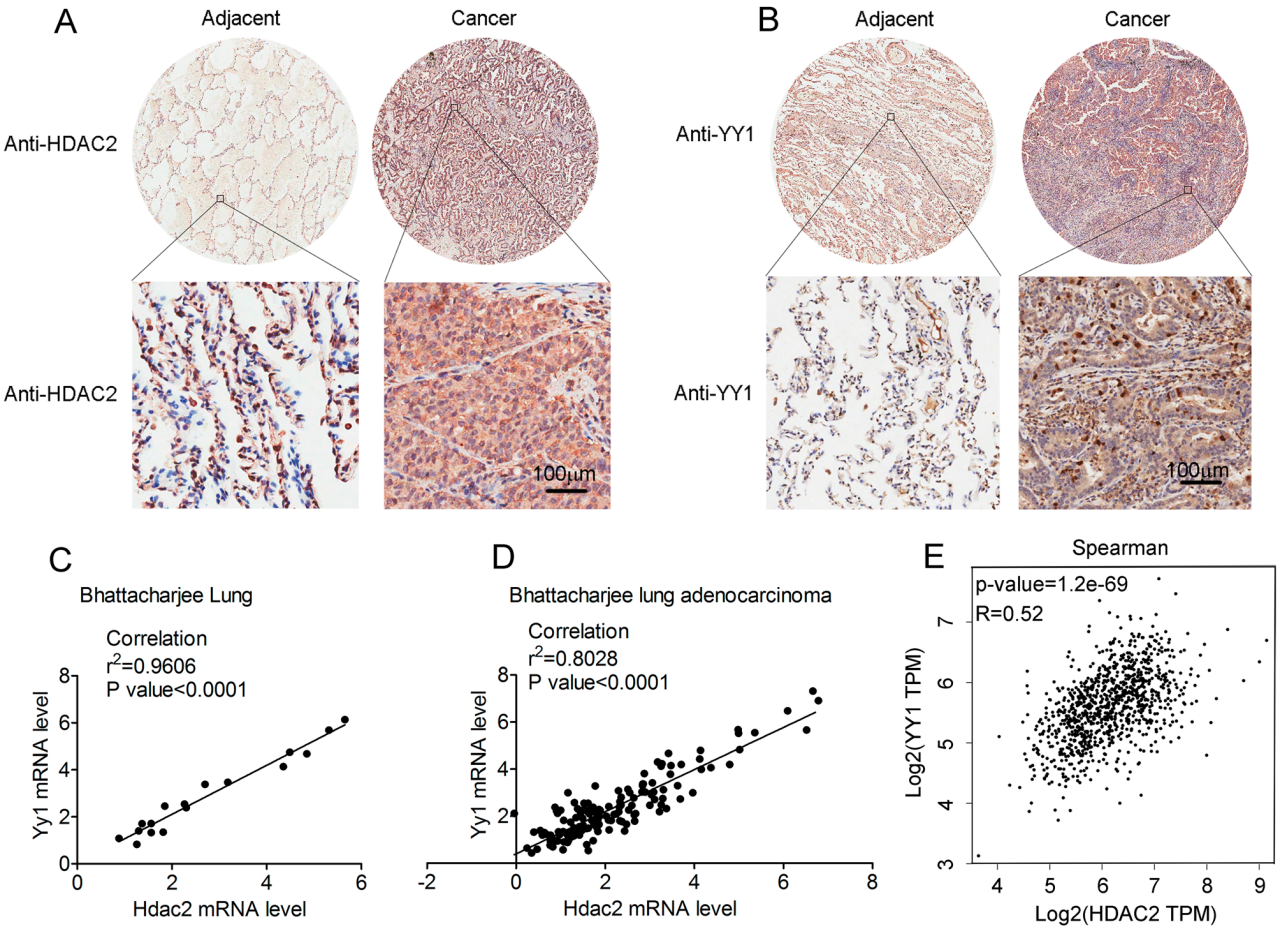
Correlation of HDAC2 and YY1 in lung adenocarcinoma tissues. (**A**, **B**) IHC of HDAC2 (**A**) and YY1 (**B**) in human lung adenocarcinoma tissues. (**C**–**E**) The correlation of HDAC2 with YY1 in lung (**C**), lung adenocarcinoma tissues (**D**, **E**) was analyzed in GEPIA.Magnification is 200-fold, and scale bar is 50 μm.

In order to examine the clinical significance of YY1 and HDAC2 in lung adenocarcinoma, we conducted an analysis of their expression levels in clinical patient samples. Our findings indicate that both YY1 and HDAC2 were significantly upregulated in cancerous samples when compared to their corresponding adjacent tissues (Fig. 6A,B). Furthermore, our examination of the TCGA database demonstrated a positive association between YY1 and HDAC2 expression in both lung (Fig. 6C,D) and adenocarcinoma tissues (Fig. 6E). These observations suggest that YY1 and HDAC2 may have crucial roles in the initiation and progression of lung adenocarcinoma.

### HDAC2 inhibits YY1 induced Cdh1 transcription

**Figure 7:**
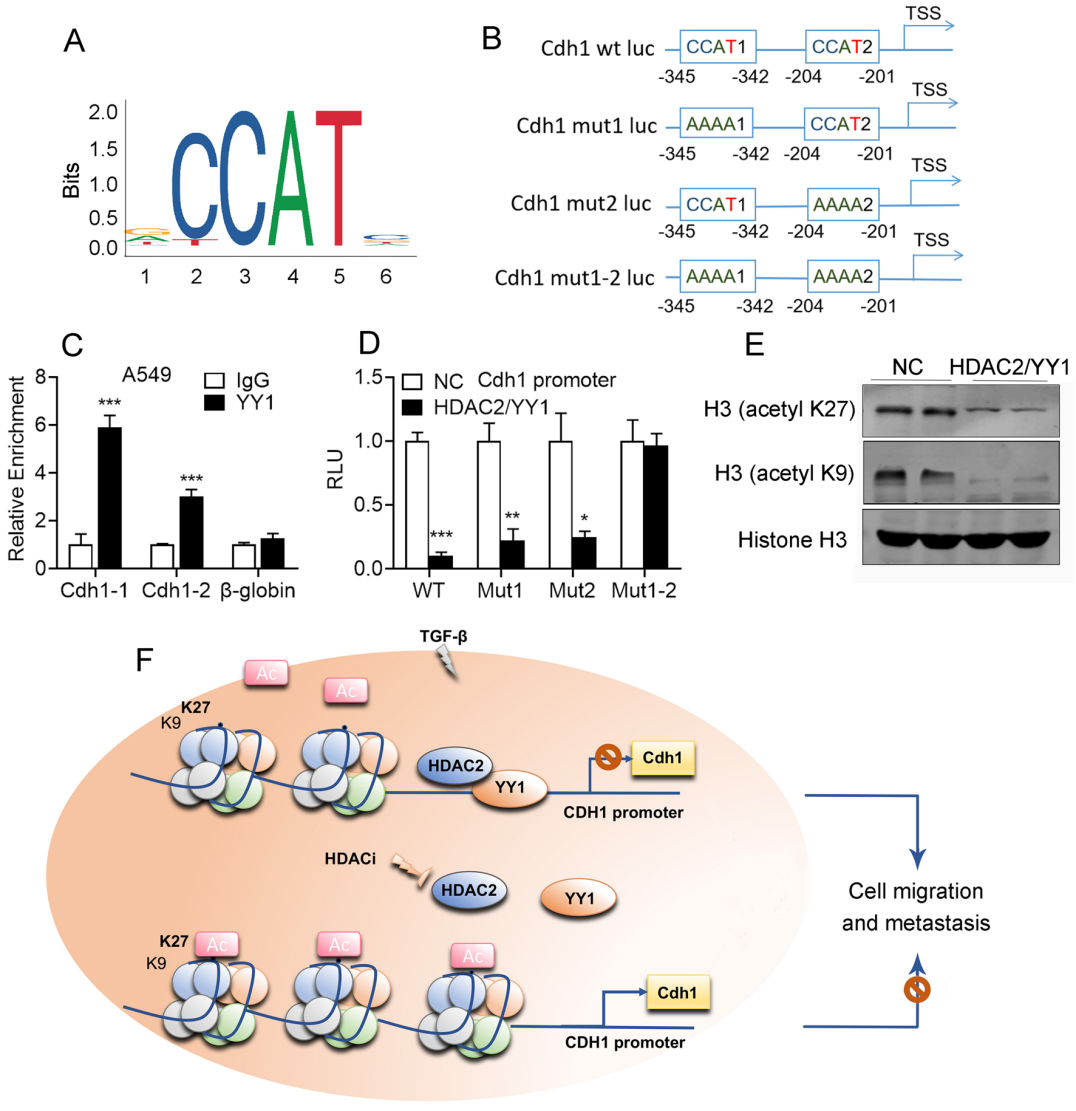
YY1 promotes Cdh1 transcriptional activity. (**A**) The binding site and sequence logo of YY1 on the promoter. (**B**) The analysis of YY1 binding site and the design of binding site mutations of homo Cdh1 promoter. (**C**) ChIP assay of YY1 on the Cdh1 promoter. (**D**) Luciferase activities of wild type (WT) and mutated Cdh1 promoter (Mut1, Mut2, Mut1-2) were determined upon HDAC2/YY1 overexpression. (**E**) The protein level of K27 and K9 acetylated Histone 3 in HDAC2/YY1 overexpressed A549 cells. (**F**) Schematics illustrating the HDAC2/YY1/Cdh1 axis in regulating TGF-β induced EMT in lung adenocarcinoma. The accumulation and interaction of HDAC2 and YY1, induced by TGF-β, result in the deacytalation of the Cdh1 promoter, leading to the inhibition of Cdh1 expression and the induction of migration in lung adenocarcinoma. Conversely, in the presence of HDACi, the disruption of the HDAC2 and YY1 interaction leads to the acytalation of the Cdh1 promoter, inducing Cdh1 expression and inhibiting migration in lung adenocarcinoma. Data are presented as mean ± SEM, and *P < 0.05, **P < 0.01, ***P < 0.001 compared with the control group. All experiments were performed at least three times.

YY1, a well-known transcription factor, plays a crucial role in regulating gene expression in diverse cellular processes, with its canonical binding sites being CCAT (Fig. 7A). Upon analyzing the promoter sequence of EMT-related genes, we identified two binding sites on the Cdh1 promoter and subsequently designed three mutated promoters with altered binding sites (Fig. 7B).The ChIP assay yielded evidence that YY1 interacts with the Cdh1 promoter, as depicted in Fig. 7C. Furthermore, YY1 was found to enhance Cdh1 promoter activity, which was impeded by mutations in the binding sites on the promoter (Fig. 7D). Our investigation also involved an analysis of the acetylation level of the canonical lysine sites of histone 3, which revealed that HDAC2 deacetylated K27 and K9 acetylation (Fig. 7E and Fig. S5). Finally, the graphic abstract of this study is presented in Fig. 7F.

## Materials and methods

### Cell culture

The HEK293T, A549, and H441 cell lines were procured from the American Type Culture Collection (Manassas, VA, USA). HEK293T cells were maintained in Dulbecco's modified Eagle's medium (DMEM, Thermo Scientific Hyclone, Rockford, IL, USA), while A549 and H441 cells were cultured in RPMI1640 medium supplemented with 10% fetal bovine serum (FBS), 100 units/ml penicillin, and 100 μg/ml streptomycin (Life Technologies Gibco, Grand Island, NY, USA). All cell lines were incubated at 37 °C in a humidified atmosphere containing 5% CO2.

### Reagents

Thermo Scientific (Gibco, Grand Island, NY, USA) was the source of RPMI1640, DMEM, Fetal bovin serum, 100 units/ml penicillin and 100 μg/ml streptomycin, and 0.25% trypsin. SinoBiological (Beijing, China) supplied the human recombinant TGF-β1. Addgene (Cambridge, MA, USA) provided the lentivirus system plasmids pCDH-CMV-MCSEF1-Puro, psPAX2, and pMD2.G. Protein-A/G sepharose was procured from Santa Cruz Biotechnology (Santa Cruz, CA), while BD Biosciences (Franklin Lakes, NJ, USA) supplied mouse anti-E-cadherin and mouse anti-N-cadherin. The Rabbit anti-Vimentin antibodies were procured from Cell Signaling Technology located in Danvers, MA, USA. The IRDye 680RD Secondary Antibodies were acquired from LI-COR Biosciences based in Nebraska, USA. The RNAiso Plus, PrimeScript™ RT Master Mix, and SYBR Green qPCR Master Mix were obtained from Takara in Beijing, China. The 10% neutral buffered formalin solution (HT501128-4L) was purchased from Sigma located in St Louis, MO, USA. The RIPA buffer and Hematoxylin/eosin staining kit were procured from Beyotime in Shanghai, China. The Rabbit anti-YY1 antibody (ab109237) and Rabbit anti-HDAC2 antibody (ab32117) were purchased from Abcam.

### Plasmid construction and RNA interference assays

The pCDH Puro-IRES-GFP vector (Addgene) was utilized to amplify and ligate the full-length open-reading frame of hHDAC2 (NM_001527.4), and hYY1 (NM_003403.5), while the pLKO.1 Vector (Addgene) was used to ligate shHDAC2 and shYY1, with primer details provided in Tables 1 and 2. HEK293T cells were employed to package the expressed lentivirus of pCDH-hHDAC2, pCDH-hYY1, shHDAC2, and shYY1 via calcium chloride transfection, followed by virus concentration, filtration, and addition to A549 lung cancer cells in the presence of 1 μg/ml polybrene for 24 h. The cells were subsequently selected with 1ug/mL puromyc.

**Table 1:** Primers used for plasmid construction.

Gene names	Forward/reverse	Sequences 5′–3′
hHDAC2 (NM_001527.4)	Forward	ATGGCGTACAGTCAAGGAGGCG
	Reverse	AATTGGTGAGACTGTCAAATTCAGGG
hYY1 (NM_003403.5)	Forward	ATGGCCTCGGGCGACACCCTCTACAT
	Reverse	CGTGGTCGAGAAGGGTCTTCTCTC

**Table 2:** Primers for realtime PCR.

Gene names	Forward/reverse	Sequences 5′–3′
hGAPDH	Forward	GAAATCCCATCACCATCTTCCAGG
	Reverse	CAGTAGAGGCAGGGATGATGTTC
hYy1	Forward	ACGGCTTCGAGGATCAGATTC
	Reverse	TGACCAGCGTTTGTTCAATGT
hHdac2	Forward	ATGGCGTACAGTCAAGGAGG
	Reverse	TGCGGATTCTATGAGGCTTCA
hCdh1	Forward	GGCACAGATGGTGTGATTACAGTC
	Reverse	AGTCTCTCTTCTGTCTTCTGAGGCCA
hCdh2	Forward	ATGCTGACGATCCCAATGC
	Reverse	GCCTTCCATGTCTGTAGCTTGA
hVimentin	Forward	GACGCCATCAACACCGAGTT
	Reverse	CTTTGTCGTTGGTTAGCTGGT
hZo1	Forward	CAACATACAGTGACGCTTCACA
	Reverse	CACTATTGACGTTTCCCCACTC
hHdac1	Forward	CCGCATGACTCATAATTTGCTG
	Reverse	ATTGGCTTTGTGAGGGCGATA
hHdac3	Forward	TCTGGCTTCTGCTATGTCAACG
	Reverse	CCCGGTCAGTGAGGTAGAAAG
hHdac4	Forward	AGCGTCCGTTGGATGTCAC
	Reverse	CCTTCTCGTGCCACAAGTCT
hHdac5	Forward	TCTTGTCGAAGTCAAAGGAGC
	Reverse	GAGGGGAACTCTGGTCCAAAG
hHdac6	Forward	AAGAAGACCTAATCGTGGGACT
	Reverse	GCTGTGAACCAACATCAGCTC
hHdac7	Forward	TGCCCAGTCCTTAATGACCAC
	Reverse	CACCTGGACGTGAGTTTTGAG
hHdac8	Forward	TCGCTGGTCCCGGTTTATATC
	Reverse	TACTGGCCCGTTTGGGGAT
hHdac9	Forward	AGTAGAGAGGCATCGCAGAGA
	Reverse	GGAGTGTCTTTCGTTGCTGAT
hHdac10	Forward	CAGTTCGACGCCATCTACTTC
	Reverse	CAAGCCCATTTTGCACAGCTC
hHdac11	Forward	ACCCAGACAGGAGGAACCATA
	Reverse	TGATGTCCGCATAGGCACAG

### RNA isolation and real-time PCR

RNA extraction was carried out by following the manufacturer's instructions^23^ using RNAiso Plus (Takara, Japan). Subsequently, the mRNA was reverse transcribed at 37 °C for 15 min using PrimeScript™ RT Master Mix (Takara, Japan). Real-time PCR was performed using the SYBR Green PCR Master Mix (Applied Biosystems, USA). The relative expression levels were determined using the ΔΔCt method of relative quantitation and normalized to human GAPDH (glyceraldehyde-3-phosphate dehydrogenase) expression. Unless otherwise specified, the data presented are derived from three independent biological replicates, each of which was assayed in triplicate. The primers used for real-time PCR are listed in Table 3.

**Table 3:** Sequences used for knockdown of target genes.

Gene names	Gene Bank number	Sequence number	Sequences 5′–3′
Homo HDAC2	NM_005195	1	AACCAGGAGATGCAGCAGAAG
		2	TGAGAACGAGAAGCTGCACCA
		3	CAACAGCAATCACAAGGCGGG
Homo YY1	NM_181659	1	AATTGCCATGTGATACTCCAG
		2	AATGCGCCAGAGATATGAAAC
		3	AAGGGATTAGACCACCTATGG

### Luciferase reporter assay

The Homo Cdh1 wt, mut1, mut2, and mut1-2 promoter sequences were cloned into the pGL3 basic firefly luciferase vector (Thermo Scientific) and subsequently transfected into 293T cells. Following a 24-h incubation period, cell lysates were prepared in reporter lysis buffer (Promega, Madison, WI, USA) and luciferase substrate was introduced. The resulting luciferase activity was measured using a luminometer (Veritas, Promega).

### Stable transfection

The lentivirus containing HDAC2, YY1, shHDAC2, and shYY1 was produced in HEK293T cells through calcium chloride transfection. The viral supernatants were collected thrice at 24-h intervals post-transfection, followed by centrifugation, filtration, and infection of lung cancer cells. The cells were then subjected to selection with puromycin for a minimum of 1–2 weeks. The efficacy of gene overexpression or knockdown was assessed through qPCR or western blotting.

### Cell lysates preparation and immunoblotting

The preparation of cell lysates and immunoblotting was conducted in accordance with previously described methods^24^. Specifically, cells were lysed using RIPA lysis buffer (Beyotime, Shanghai) supplemented with protease and phosphotase inhibitors and PMSF. The resulting lysates were collected and subjected to centrifugation at 12,000 rpm for 15 min at 4 °C. The soluble protein was then quantified using a BCA quantification kit (Beyotime, Shanghai), and protein samples were subsequently electrophoresed on SDS-PAGE and transferred onto nitrocellulose membranes (Pall, Amersham). The membranes were blocked using 5% defatted milk in TBST buffer supplemented with 0.1% Tween-20. Following this, the membranes were subjected to an overnight incubation with the suitable primary antibodies at 4 °C. The membranes were then rinsed with TBST and exposed to secondary antibodies. Protein bands were detected using super signal reagents. Antibodies were diluted in accordance with the manufacturer's instructions. β-actin was utilized as a loading control.

### Co-Immunoprecipitation

The present study conducted an assay in accordance with a prior report^25^. Specifically, cellular lysis was carried out in ice-cold RIPA buffer supplemented with 1 mM PMSF and 10uM Protein Kinase Inhibitor on ice for 30 min. Subsequently, the insoluble fraction was removed via centrifugation at 4℃ for 15 min at 12,000 rpm, and the soluble protein was quantified using a BCA quantification kit and pre-cleared using protein-A/G sepharose. Immunoprecipitation was performed by incubating the aforementioned lysates with either anti-HDAC2 or anti-YY1 primary antibody at 4 °C for 8–12 h, with normal IgG serving as the negative control. The mixture of antibody-lysates was supplemented with protein-A/G sepharose and incubated at 4 °C for 2–4 h. The resulting antigen–antibody–sepharose complex was washed with PBS at 4 °C every 5 min. Subsequently, the complex was collected and heated in 1 × loading buffer at 95 °C for 5 min, followed by immunoblotting of the eluted proteins. The manufacturer's instructions were followed to determine the appropriate quantity of specific antibody for this assay.

### Wound healing assays

5 × 10^5^ cells were seeded in 35-mm culture dish. 24 h later, wounds were incised in the middle area of the confluent cell culture, followed by addition of fresh medium after carefully washing off the detached cells. Images were taken of the wounded area using Nikon digital camera at 4 × magnification at 0, 24, and 48 h.

#### Transwell assays

The present study conducted an assay in accordance with a prior report^26^. Specifically, 5 × 10^4^ cells were suspended in culture medium containing 0.5% FBS and subsequently seeded into the upper well of a transwell chamber (Corning Costar, Thermo fisher, NY), while the lower well was filled with culture medium containing 10% FBS as a chemoattractant. Following incubation for 16 h at 37 °C in the presence of 5% CO_2_, non-migrated cells were removed from the upper surface. The migrated cells were then fixed with alcohol and stained with H/E. The total number of migrated cells was determined by counting the cells using a Nikon digital camera at 200× magnification.

#### Xenograft mouse model

Female athymic BALB/c nude mice (4 weeks) were purchased from Gempharmatech (Nanjing, China). All animal experiments were approved by Animal Ethics Committee of Zhengzhou University (Approval Number: ZZU-LAC20230616^18^). All methods were performed in accordance with the relevant guidelines and regulations. Also all methods are reported in accordance with ARRIVE guidelines. Mice were allocated to experimental groups randomly. 5 × 10^6^ cells were injected into mice through tail vein. 4–6 weeks later, animals were euthanized and tissues were collected for tumor counting and HE staining.

#### STRING analysis

The analysis of the interaction among proteins are performed by STRING online software, the website is https://string-db.org/.

#### Online database

The present study assessed the expression levels of HDACs in lung tumor and adjacent tissues, which were categorized as “high” or “low” based on their expression levels relative to the median value of all samples. The survival rate of the “high” and “low” expression groups was analyzed using the log-rank test. The correlation between gene expression and survival was evaluated using Kaplan–Meier plots generated by Gene Expression Profiling Interactive Analysis (GEPIA) in the http://gepia.cancer-pku.cn/^27^.

### Ethical approval

The lung cancer tissue chip (Hlug-Ade 060PG-01) was purchased from Shanghai Outdo Biotechnology Company and the clinical information was listed in Table 4.

**Table 4:** Clinical information of the lung cancer tissue chip (Hlug-Ade 060PG-01).

Tissue type	Tissue code	Organ	Gender	Age	Primary organ	Metastasis	Pathological classification	Histological grade	Tumor volume
Lung/adjacent	RRsLug0906A0726	Lung	Female	66	No	Yes	Adenocarcinoma	I	3 × 2.5 × 2 cm
Lung/adjacent	RRsLug0906A0722	Lung	Female	68	No	Yes	Adenocarcinoma	I	2.5 × 2.5 × 2 cm
Lung/adjacent	E05A0759	Lung	Female	69	No	Yes	Adenocarcinoma	I	2 × 1.5 × 2 cm
Lung/adjacent	RRsLug0801A0450	Lung	Female	58	No	Yes	Adenocarcinoma	I–II	2.5 × 2.5 × 2 cm
Lung/adjacent	E05A0796	Lung	Female	63	No	Yes	Adenocarcinoma	I–II	3 × 2 × 1 cm
Lung/adjacent	CRsLug0612A0232	Lung	Female	71	No	Yes	Adenocarcinoma	II	3 × 2.5 × 2 cm
Lung/adjacent	CRsLug0612A0238	Lung	Male	51	No	Yes	Adenocarcinoma	II	3 × 3 × 2 cm
Lung/adjacent	CRsLug0702A0260	Lung	Female	56	No	Yes	Adenocarcinoma	II	Diameter 5 cm
Lung/adjacent	CRsLug0702A0264	Lung	Female	30	No	Yes	Adenocarcinoma	II	3.5 × 3.5 × 2 cm
Lung/adjacent	CRsLug0706A0369	Lung	Male	42	No	Yes	Adenocarcinoma	II	2.5 × 2.5 × 2 cm
Lung/adjacent	CRsLug0707A0372	Lung	Male	63	No	Yes	Adenocarcinoma	II	3 × 3 × 2.5 cm
Lung/adjacent	CRsLug0709A0392	Lung	Female	57	No	Yes	Adenocarcinoma	II	3.5 × 3 × 3 cm
Lung/adjacent	CRsLug0709A0399	Lung	Female	51	No	Yes	Adenocarcinoma	II	4 × 3 × 3 cm
Lung/adjacent	CRsLug0711A0441	Lung	Female	60	No	Yes	Adenocarcinoma	II	3 × 2 × 1.5 cm
Lung/adjacent	CRsLug0804A0507	Lung	Female	61	No	Yes	Adenocarcinoma	II	2 × 1.5 × 1 cm
Lung/adjacent	CRsLug0806A0539	Lung	Female	50	No	Yes	Adenocarcinoma	II	Diameter 3 cm
Lung/adjacent	CRsLug0806A0542	Lung	Male	70	No	Yes	Adenocarcinoma	II	4 × 3 × 3 cm
Lung/adjacent	CRsLug0812A0627	Lung	Male	59	No	Yes	Adenocarcinoma	II	4 × 4 × 3 cm
Lung/adjacent	CRsLug0812A0629	Lung	Male	40	No	Yes	Adenocarcinoma	II	3 × 2.5 × 2 cm
Lung/adjacent	CRsLug0902A0648	Lung	Female	62	No	Yes	Adenocarcinoma	II	3 × 2.5 × 2 cm
Lung/adjacent	CRsLug0612A0230	Lung	Female	52	No	Yes	Adenocarcinoma	II–III	4 × 3.5 × 3 cm
Lung/adjacent	CRsLug0711A0433	Lung	Female	63	No	Yes	Adenocarcinoma	II–III	3.5 × 2.5 × 3.5 cm
Lung/adjacent	CRsLug0711A0438	Lung	Male	60	No	Yes	Adenocarcinoma	II–III	3 × 3 × 2.5 cm
Lung/adjacent	CRsLug0806A0559	Lung	Female		No	Yes	Adenocarcinoma	II–III	Diameter 3 cm
Lung/adjacent	NRsLug0312A0002	Lung	Female	37	No	Yes	Adenocarcinoma	III	4 × 3 × 3 cm
Lung/adjacent	CRsLug0512A0149	Lung	Male	67	No	Yes	Adenocarcinoma	III	6 × 6 × 5 cm
Lung/adjacent	CRsLug0709A0388	Lung	Female	63	No	Yes	Adenocarcinoma	III	3.5 × 2.5 × 1.5 cm
Lung/adjacent	CRsLug0812A0623	Lung	Male	67	No	Yes	Adenocarcinoma	III	6 × 6 × 5 cm
Lung/adjacent	CRsLug0902A0636	Lung	Male	67	No	Yes	Adenocarcinoma	III	5 × 6 × 4 cm
Lung/adjacent	CRsLug0903A0657	Lung	Male	62	No	Yes	Adenocarcinoma	III	4 × 3.5 × 3 cm

### Statistical analysis

The statistical analysis was from more than three independent experiments performed in duplicates or up to three parallel controls. For experiments with two groups, statistical signifificance was determined by Student’s t-test. For experiments with more than three groups, statistical analyses were performed with analysis of variance followed by post hoc pairwise comparisons. The data shown are means ± SEM. The *P*-value of less than 0.05 was considered statistically significant. The *P*-values were designated as *, *P* < 0.05, **, *P* < 0.01, ***, *P* < 0.001. ns, non significant.

## Discussion

HDAC inhibitors have demonstrated potential in regulating the progression of cancer cells. A multitude of HDAC inhibitors, such as vorinostat(SAHA)^28^, romidepsin^29^, belinostat, panobinostat, and entinostat, have been associated with the management of cancer cell progression^30^. Prior studies have indicated that HDACi (histone deacetylase inhibitors) impede cancer progression by influencing diverse cellular processes, including tumor growth, programmed cell death, metastasis, and angiogenesis^31^. Further investigation is necessary to comprehensively elucidate the precise molecular mechanisms underlying the control of cancer progression by HDAC inhibitors. Although more than ten HDACs are present in mammalian cells, there is currently no evidence indicating which one is pivotal in the migration of lung adenocarcinoma. This study has provided insight into the heightened expression and induction of HDAC2 during the process of lung adenocarcinoma, which has captured our attention. Furthermore, we have identified that two HDAC inhibitors with distinct structures, TSA and NaBu^32^, impede lung adenocarcinoma migration via HDAC2 by interacting with YY1, a transcription factor, and deacetylating Cdh1, a tumor suppressor gene^33^. The downregulation of Cdh1 is often observed in cancer cells undergoing epithelial-mesenchymal transition (EMT), a process that enables cancer cells to acquire a more motile and invasive phenotype^34^.

It is noteworthy that the involvement of HDAC2 in tumorigenesis varies across different cancer types and is often complex and even contradictory. For instance, in glioblastoma tumorigenesis, HDAC2 knockdown has been shown to impede tumor-sphere formation and proliferation by upregulating miR-3189-mediated GLUT3^35^.In contrast, HDAC2 functions as a metastasis suppressor in colorectal cancer by impeding EMT and the expression of H19 and MMP14^36^. These observations indicate that HDAC2 exhibits disparate roles in various cancer types. Nevertheless, the precise functional mechanism of HDAC2 in lung adenocarcinoma migration remains elusive, necessitating a comprehensive comprehension of its role in lung adenocarcinoma patients.

Multiple studies have reported on the interplay between HDAC2 and YY1, but this complex exhibits divergent functions in different tissues. For example, the physical interaction between FKBP25 and histone deacetylases HDAC1 and HDAC2, as well as the HDAC-binding transcriptional regulator YY1, results in the modification of YY1's DNA-binding activity^37^. In clear cell renal cell carcinoma, the YY1/HDAC2 complex reduces the expression of YTHDC1, which modulates the sensitivity of ccRCC to sunitinib by targeting the ANXA1-MAPK pathway^38^. Additionally, the YY1/HDAC2 signaling pathway is crucial in regulating cell proliferation in human colorectal cancer^39^. Furthermore, a recent investigation has uncovered the indispensable role of the HDAC2/YY1 complex in lung adenocarcinoma metastasis.

This study illuminates the intricate interplay among diverse molecular pathways in cancer metastasis and emphasizes the significance of devising targeted therapies that can impede these pathways and forestall cancer dissemination. Nevertheless, it is crucial to acknowledge that this study was executed in vitro, and additional research is imperative to authenticate these findings in vivo and in human clinical trials.

## Supplementary Information

Supplementary Figures.

Supplementary Information 2.

## Data Availability

The datasets used and/or analysed during the current study available from the corresponding author on reasonable request.
