# Correction: Histone deacetylase inhibitors inhibit lung adenocarcinoma metastasis via HDAC2/YY1 mediated downregulation of Cdh1

**DOI:** 10.1038/s41598-025-23928-6

**Published:** 2025-10-28

**Authors:** Dongmei Wang, Yixiao Yang, Yuxiang Cao, Meiyao Meng, Xiaobo Wang, Zhengxun Zhang, Wei Fu, Shichao Duan, Liming Tang

**Affiliations:** 1 Department of Gastrointestinal Surgery, The Affiliated Changzhou, No. 2 People’s Hospital of Nanjing Medical University, Changzhou, 213004 Jiangsu China; 2 Changzhou Medical Center of Nanjing Medical University, Changzhou, 213004 Jiangsu China; 3 Institute of Burn Research, The First Affiliated Hospital, State Key Lab of Trauma, Burn and Combined Injury, Chongqing Key Laboratory for Disease Proteomics, Third Military Medical University (Army Medical University), Chongqing, 400038 China; 4 Shanghai Key Laboratory of Regulatory Biology, Institute of Biomedical Sciences and School of Life Sciences, East China Normal University, Shanghai, 200241 China; 5 Henan Provincial Chest Hospital, Zhengzhou, 450000 Henan China; 6 Henan Provincial People’s Hospital, Henan Eye Hospital, Henan Eye Institute, Zhengzhou University People’s Hospital, Henan University People’s Hospital, Zhengzhou, 450003 Henan China

Correction to: *Scientific Reports* 10.1038/s41598-023-38848-6, published online 26 July 2023

The original version of this Article contained an error in Figure 1, where during figure assembly, the “TGF-β” panel in Figure 1A was mistakenly a duplication of the “shNC” panel from Figure 3C.

**Fig. 1:**
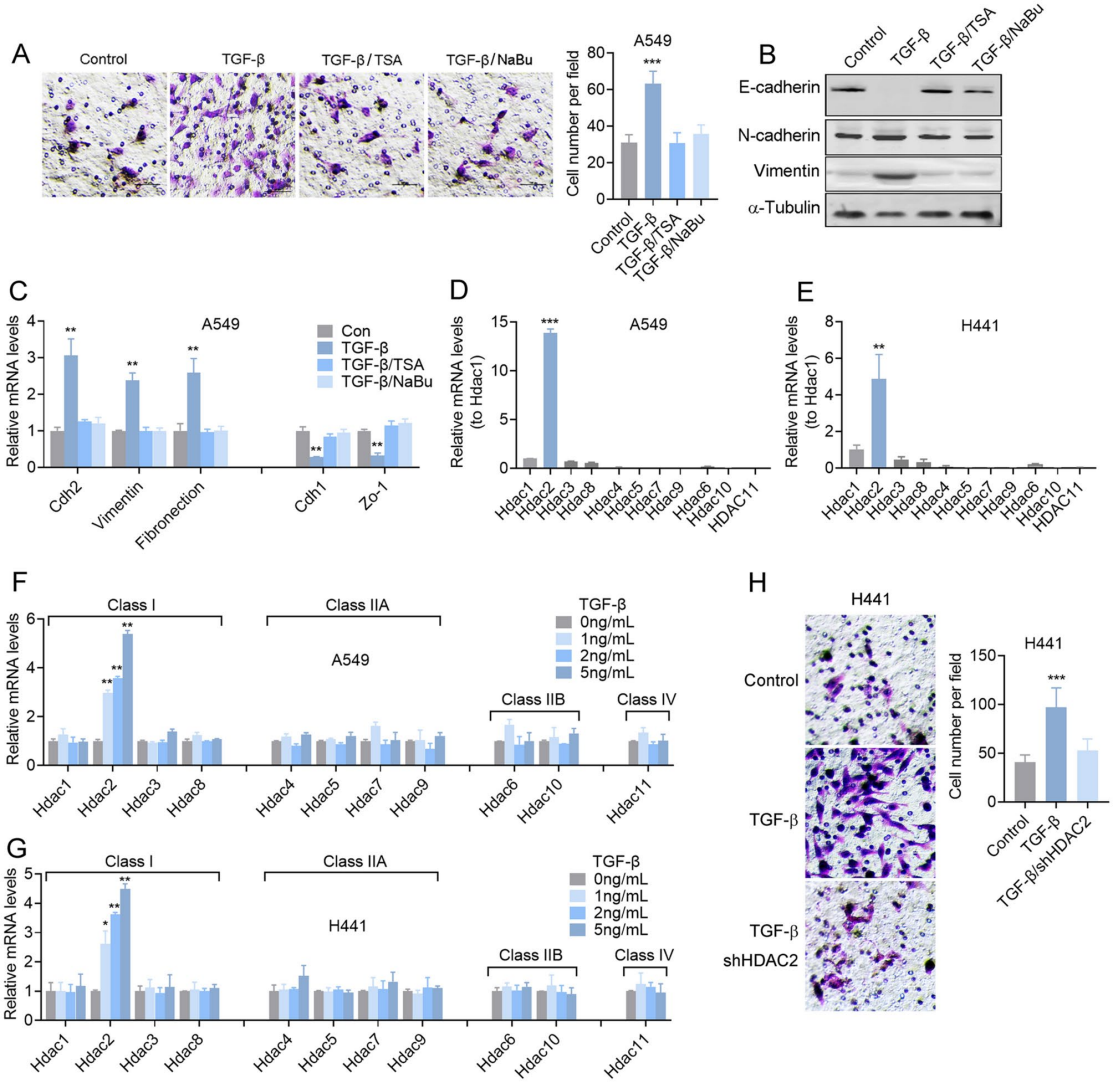
HDAC inhibitors suppress TGF-β-induced EMT of lung adenocarcinoma cells via HDAC2. (**A**) Transwell assay and (**B**) immunoblotting of A549 lung adenocarcinoma cells pretreated with 5 ng/mL TGF-β in the presence of 20 ng/mL TSA or 2 mM NaBu for 48 h. (**C**) Relative mRNA levels of EMT related molecules in A549 cells pretreated with 5 ng/mL TGF-β in the presence of 20 ng/mL TSA or 2 mM NaBu for 24 h. Relative mRNA levels of Hdacs in A549 (**D**) and H441 (**E**) lung adenocarcinoma cells. Relative mRNA levels of Hdacs in A549 (**F**) or H441 (**G**) cells pretreated with different doses of TGF-β for 24 h. (**H**) Transwell assay of shHDAC2 A549 cells pretreated with 5 ng/mL TGF-β. Magnification is 200-fold, and the scale bar is 50 μm. Data are presented as mean ± SEM, and **P < 0.01, ***P < 0.001 compared with the control group. All experiments were performed at least three times.

The original Figure 1 and its accompanying legend appear below.

The original Article has been corrected.

