# Supplementary material for: Histone deacetylase inhibitors inhibit lung adenocarcinoma metastasis via HDAC2/YY1 mediated downregulation of Cdh1
Source: Sci Rep. 2023 Jul 26;13:12069. doi: 10.1038/s41598-023-38848-6 (PMC10372082; doi:10.1038/s41598-023-38848-6)
Supplement: Supplementary file 1 — Supplementary Figures. [file 41598_2023_38848_MOESM1_ESM.docx]

Fig. S1


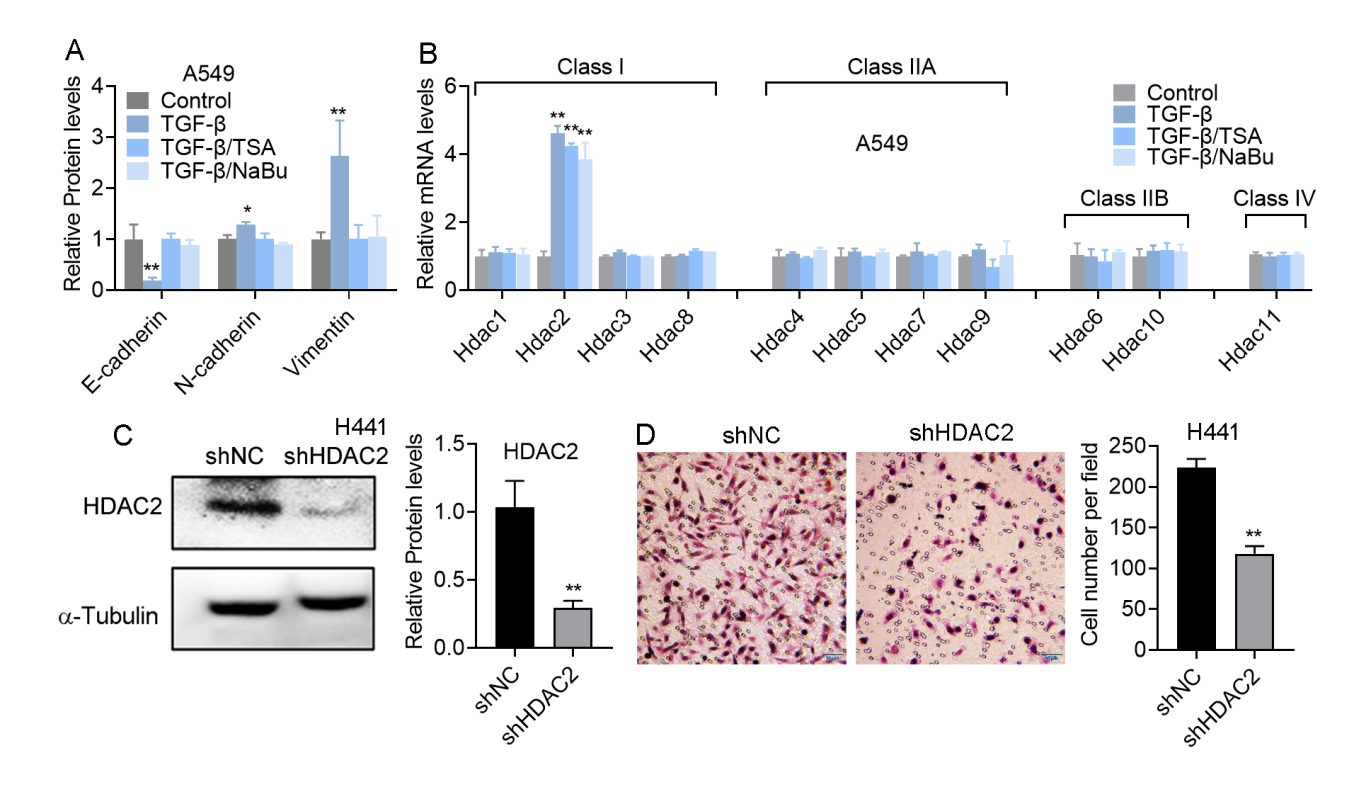


Fig. S1. A. Relative protein levels of immunoblotting of A549 lung adenocarcinoma cells pretreated with 5 ng/mL TGF-β in the presence of 20 ng/mL TSA or 2 mM NaBu for 48 hours. B. Relative mRNA levels of Hdacs of A549 cells pretreated with 5 ng/mL TGF-β in the presence of 20 ng/mL TSA or 2 mM NaBu for 24 hours. C. Immunobloting and relative protein levels of shHDAC2 H441 lung adenocarcinoma cells. C. Cell migration of shHDAC2 H441 lung adenocarcinoma cells. Magnification is 200-fold, and scale bar is 50 μm. Data are presented as mean ± SEM, and *p < 0.05, **p < 0.01 compared with the control group. All experiments were performed at least three times.

Fig. S2


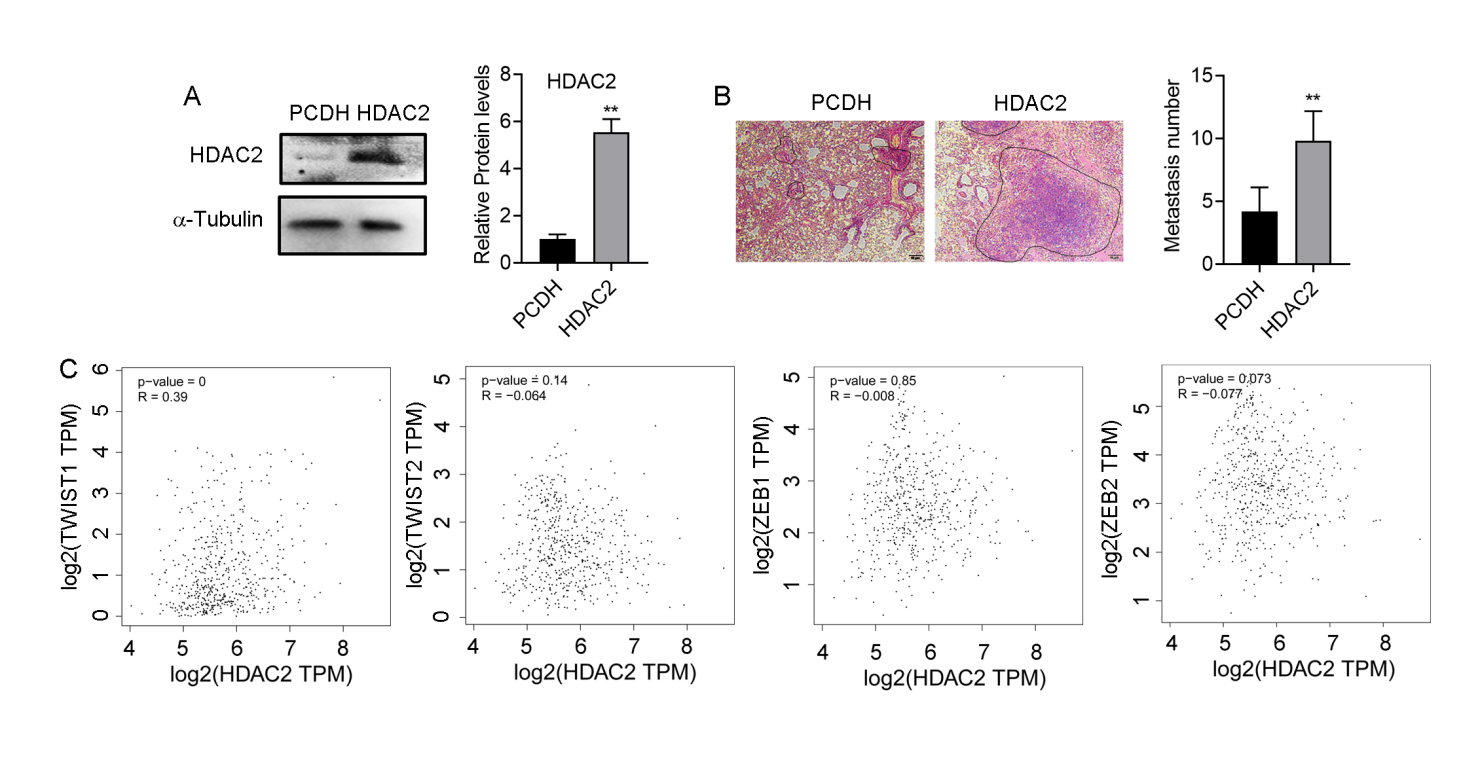


Fig. S2. A. Immunobloting and relative protein levels of HDAC2 overexpressed H441 lung adenocarcinoma cells. B. HE staining of lung tissues (left) and lung metastasis (right)of nude mice injected with HDAC2 overexpressed A549 cells. C. The correlation of HDAC2 with EMT-related transcription factors. Magnification is 100-fold, and scale bar is 50 μm. Data are presented as mean ± SEM, and **p < 0.01 compared with the control group. All experiments were performed at least three times.


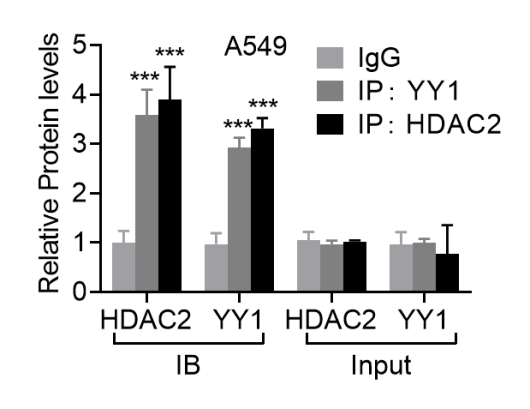
Fig. S3

Fig. S3. A. Relative protein levels of immunoblotting and co-immunoprecipitation of endogenous HDAC2 and YY1 in A549 cells. Data are presented as mean ± SEM, and ***p < 0.001 compared with the control group. All experiments were performed at least three times.


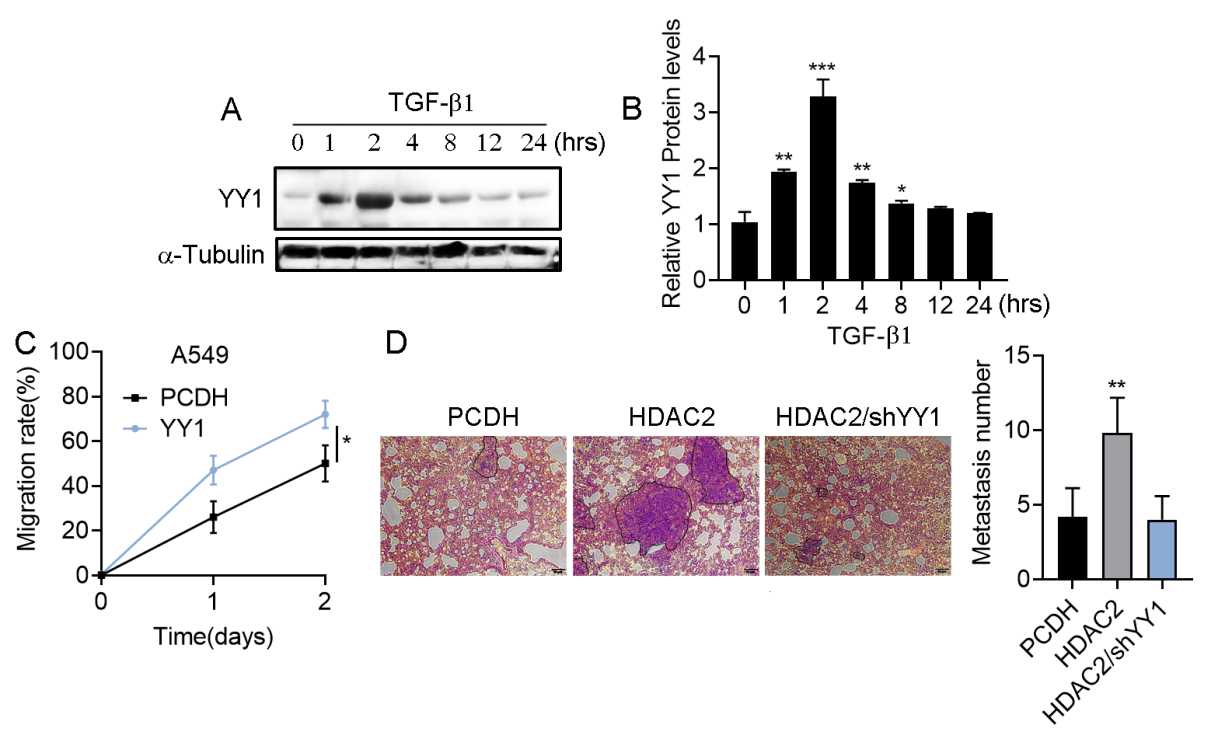
Fig. S4

Fig. S4. A. B. Immunoblotting(A) and relative protein levels (B) of the immunobloting of endogenous YY1 in A549 cells with time-dependent treatment of TGF-β. C. The migration rate of YY1 overexpressed A549 cells detected by wound healing assay. D. HE staining of lung tissues (left) and lung metastasis (right) of nude mice injected with HDAC2 overexpressed with or without YY1 of A549 cells. Data are presented as mean ± SEM, and *p < 0.05，**p < 0.01，***p < 0.001 compared with the control group. All experiments were performed at least three times.


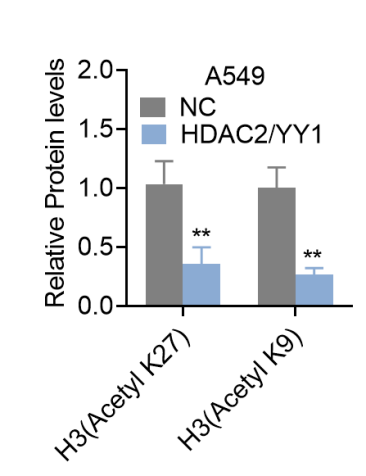


Fig. S5

Fig. S5. A. Relative protein levels of immunoblotting of Acetylyl K27 and K9 of Histone3 in HDAC2 and YY1 overexpressed A549 cells. Data are presented as mean ± SEM, and **p < 0.01 compared with the control group. All experiments were performed at least three times.
