# Supplementary figures and images for: Histone deacetylase inhibitors inhibit lung adenocarcinoma metastasis via HDAC2/YY1 mediated downregulation of Cdh1
Source: Sci Rep. 2023 Jul 26;13:12069. doi: 10.1038/s41598-023-38848-6 (PMC10372082; doi:10.1038/s41598-023-38848-6)

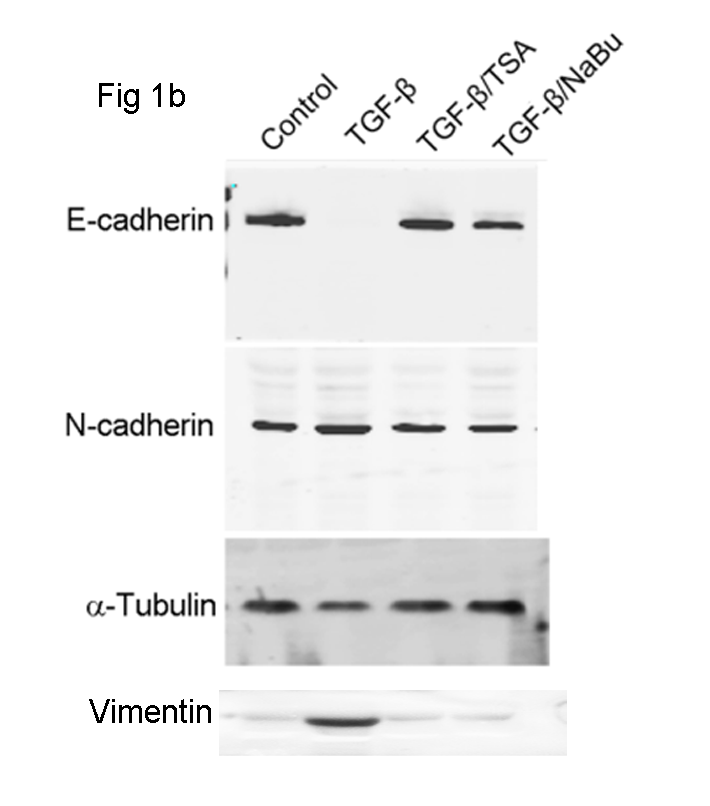
Fig1b


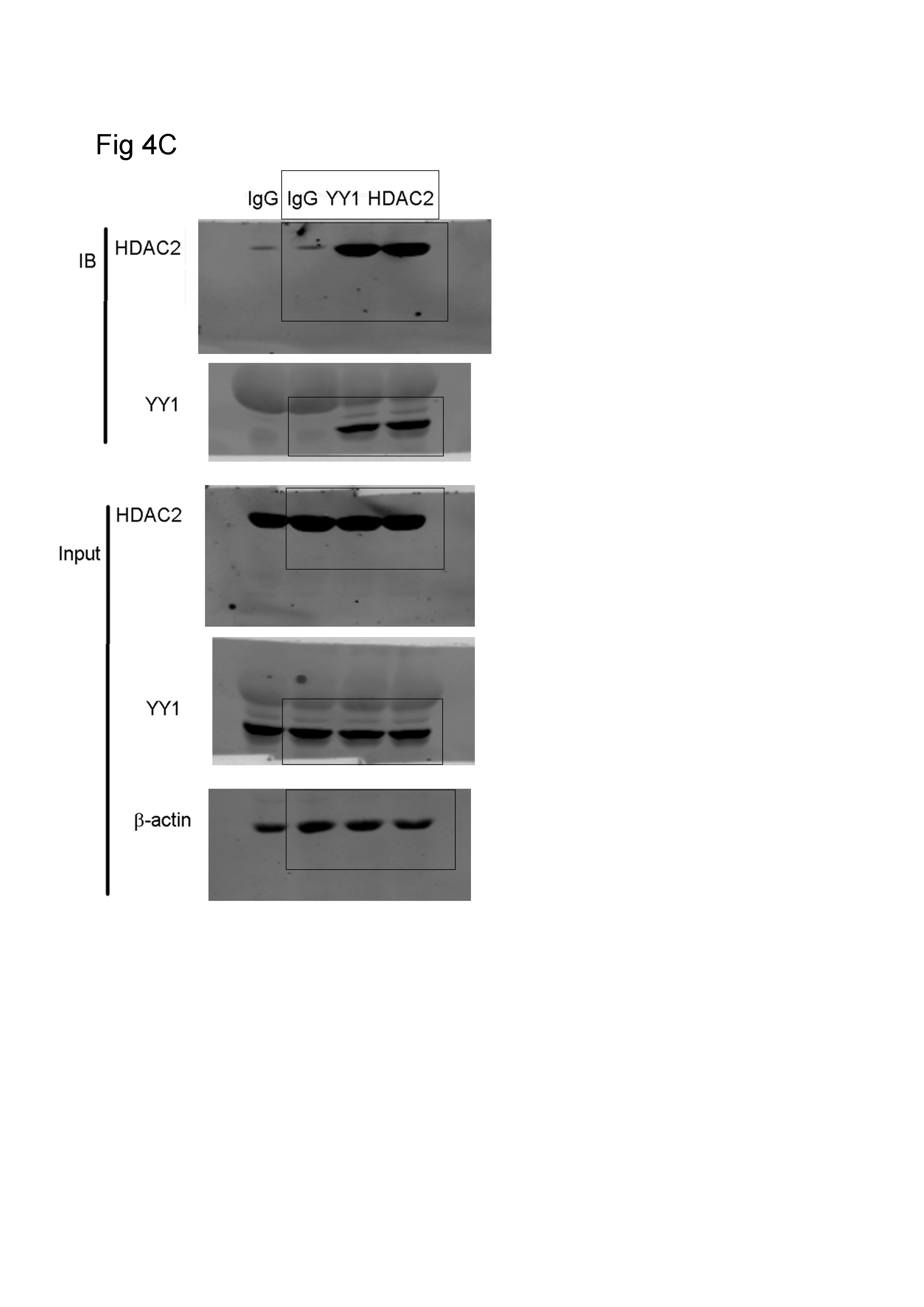


Fig 4c


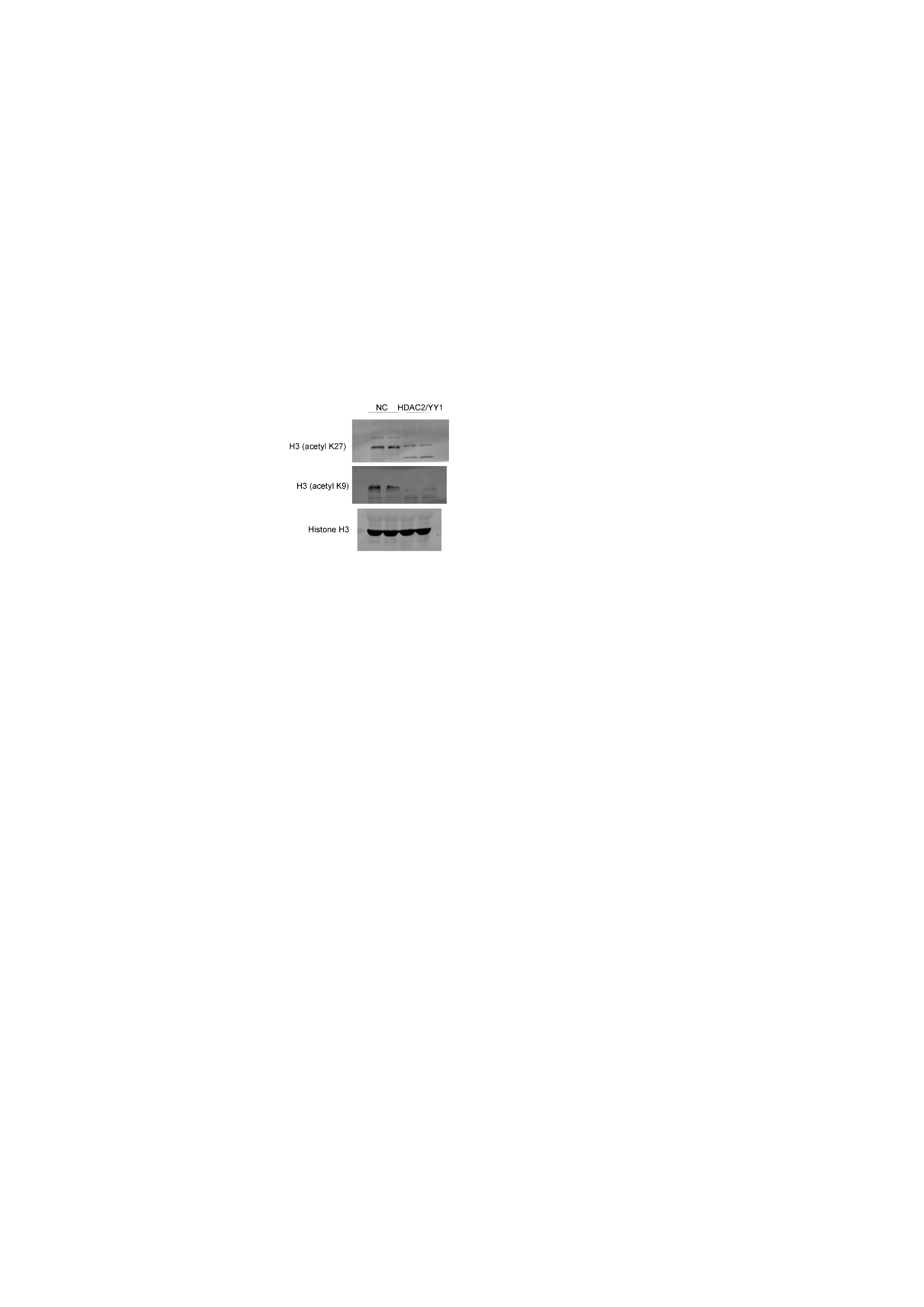
Fig 7e


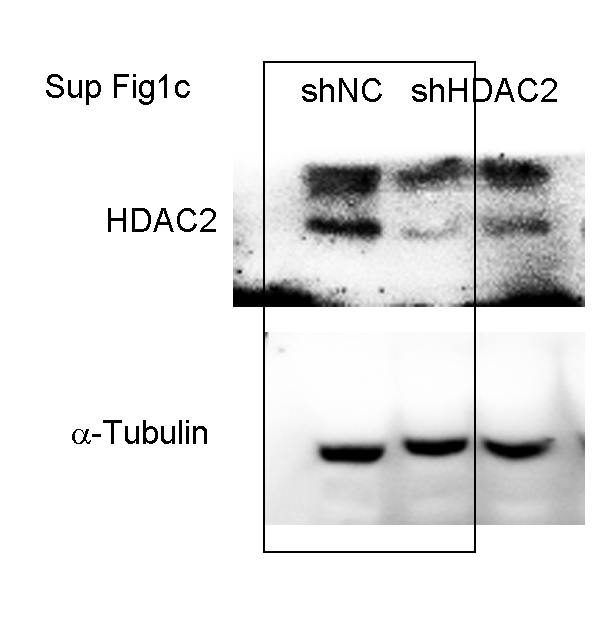


Sup Fig 1c


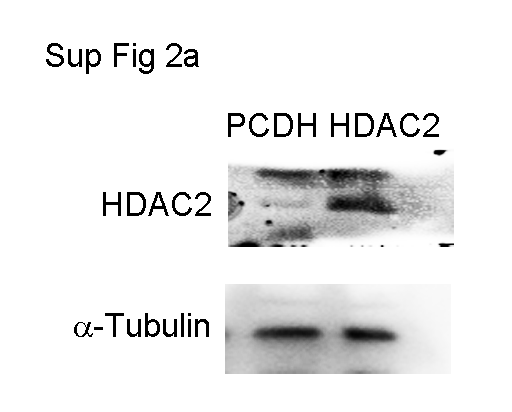


Sup Fig 2a


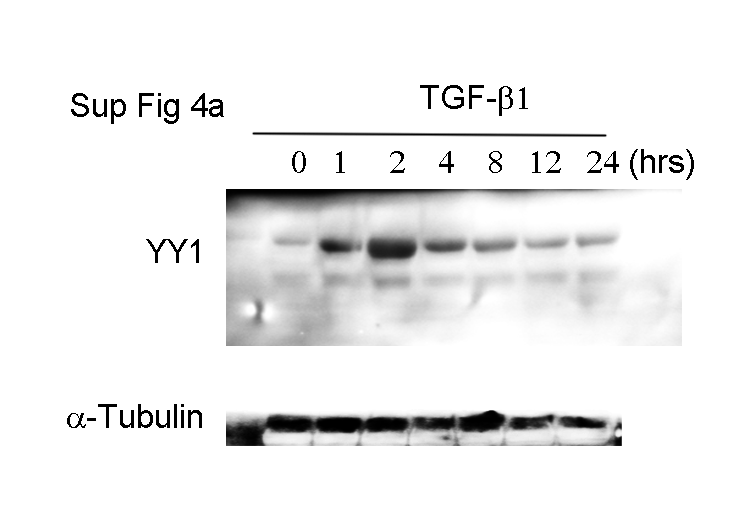


Sup Fig 4a

Supplement: Supplementary file 2 — Supplementary Information 2. [file 41598_2023_38848_MOESM2_ESM.docx]
